# Fit Accuracy of Removable Partial Denture Frameworks Fabricated with CAD/CAM, Rapid Prototyping, and Conventional Techniques: A Systematic Review

**DOI:** 10.1155/2021/3194433

**Published:** 2021-09-06

**Authors:** Naseer Ahmed, Maria Shakoor Abbasi, Sara Haider, Nimra Ahmed, Syed Rashid Habib, Sara Altamash, Muhammad Sohail Zafar, Mohammad Khursheed Alam

**Affiliations:** ^1^Prosthodontics Unit, School of Dental Sciences, Health Campus, Universiti Sains Malaysia, Kubang Kerian, 16150 Kota Bharu, Kelantan, Malaysia; ^2^Department of Prosthodontics, Altamash Institute of Dental Medicine, Karachi 75500, Pakistan; ^3^Dow Dental College, Dow University of Health Sciences, Karachi 74200, Pakistan; ^4^Department of Prosthetic Dental Sciences, College of Dentistry, King Saud University, Riyadh 11545, Saudi Arabia; ^5^Department of Orthodontics, Altamash Institute of Dental Medicine, Karachi 75500, Pakistan; ^6^Department of Restorative Dentistry, College of Dentistry, Taibah University, Al Madinah, Al Munawwarah 41311, Saudi Arabia; ^7^Department of Dental Materials, Islamic International Dental College, Riphah International University, Islamabad 44000, Pakistan; ^8^Department of Preventive Dentistry, College of Dentistry, Jouf University, Sakaka, Al Jouf 72345, Saudi Arabia

## Abstract

**Objective:**

Analyzing and comparing the fit and accuracy of removable partial denture (RPDs) frameworks fabricated with CAD/CAM and rapid prototyping methods with conventional techniques.

**Materials and Methods:**

The present systematic review was carried out according to PRISMA guidelines. The search was carried out on PubMed/MEDLINE, Cochrane collaboration, Science direct, and Scopus scientific engines using selected MeSH keywords. The articles fulfilling the predefined selection criteria based on the fit and accuracy of removable partial denture (RPD) frameworks constructed from digital workflow (CAD/CAM; rapid prototyping) and conventional techniques were included.

**Results:**

Nine full-text articles comprising 6 in vitro and 3 in vivo studies were included in this review. The digital RPDs were fabricated in all articles by CAD/CAM selective laser sintering and selective laser melting techniques. The articles that have used CAD/CAM and rapid prototyping technique demonstrated better fit and accuracy as compared to the RPDs fabricated through conventional techniques. The least gaps between the framework and cast (41.677 ± 15.546 *μ*m) were found in RPDs constructed through digital CAD/CAM systems.

**Conclusion:**

A better accuracy was achieved using CAD/CAM and rapid prototyping techniques. The RPD frameworks fabricated by CAD/CAM and rapid prototyping techniques had clinically acceptable fit, superior precision, and better accuracy than conventionally fabricated RPD frameworks.

## 1. Introduction

The accurate fit of removable partial denture (RPD) is a key component of the removable prosthodontics. Conventionally fabricated RPD can be time consuming, and their misfit has been identified as one of the chief complaints of RPD wearers. Thus, various other methods are developed and employed for the fabrication of RPDs, which are less extensive, and work effortlessly with better fit and accuracy [1]. In recent years, innovative technology such as computer-aided designing and computer-aided manufacturing (CAD/CAM) and rapid prototyping (RP) techniques has revolutionized the specialty of prosthodontics, with its use not only in removable dentures but also as extensively as for maxillofacial/craniofacial prostheses [2]. The CAD/CAM-fabricated prostheses, restorations, and devices are utilized in almost all branches of dentistry such as restorative (inlays; onlays), orthodontics (invisible aligners), and prosthetic dental sciences (ceramic veneers; single/multiple crowns; fixed partial dentures; removable complete/partial prostheses) [2, 3].

The RP have also been used to fabricate RPDs and are believed to improve the quality of fit in RPD frameworks [3]. RP constructs RPDs automatically and quickly with high accuracy making them more comfortable and acceptable to the patients. The CAD/CAM uses subtractive manufacturing such as milling techniques while RP utilizes additive manufacturing like three-dimensional (3D) printing, selective laser sintering (SLS), selective laser melting (SLM), and selective laser stereolithography (SLA) [4]. The components manufactured for RPDs must satisfy the functional and biomechanical needs such as retention, stability, support, reciprocation, encirclement, and passivity [5]. The biomechanical needs of the RPDs should accommodate the movements of prosthesis during function without exerting compulsive stresses on the abutment teeth, rigidity of the major connectors, with occlusal rests directing the occlusal forces along the long axis of the teeth and guide planes for enhancing stability [3, 6].

The 3D printing technique has opened a new method of construction of RPD which has clinically presented better accuracy and fit. It is widely being used because of its time effectiveness for the construction of prostheses. It also provides a clean workplace for the technician, not having to deal with plaster or dust [6]. In recent times, digital scans have also been introduced in dentistry and have been shown to have better trueness than the conventional impressions [7]. Digital scans have prevented the need for placing impression material in the mouth, which causes gag reflex and claustrophobia in a great number of patients. This highly advanced technology is acceptable for patients who avoid the use of conventional impressions [7]. Most commonly used material to manufacture RPDs with SLM is cobalt-chromium alloy (Co-Cr). The Co-Cr is commonly used due to its physical properties fulfilling the requirements of RPDs including accurate fit, excellent mechanical properties, easy to clean, and does not intrude with tongue space [8].

Both methods of manufacturing, i.e., conventional and digital, yields satisfactory results, but the digital method of construction of RPDs is less time consuming, with simplified technique and minimal chances of laboratory or clinical errors and better accuracy and fit [5, 9]. Minimum chair side time is needed to evaluate the fit of digitally manufactured RPDs. Trials have been conducted on patients to check the fitting of the dentures fabricated by CAD/CAM and RP techniques [10], and the results are encouraging with regard to patient's satisfaction and optimal biomechanical performance. Hence, this newest technology of fabrication of RPDs is broadly accepted and used all over the globe. The aim of the present systematic review was to analyze the available literature pertaining to the experimental fit accuracy of digitally designed and fabricated RPDs versus conventionally fabricated RPDs.

## 2. Materials and Methods

### 2.1. Research Question

In this systematic review, we followed preferred reporting items for systematic review and meta-analysis (PRISMA) guidelines and Participants Intervention Control Outcomes (PICO) protocol. The focused question was “Does digitally fabricated (CAD/CAM and RP) RPDs frameworks (connectors, retainer, reciprocal components, and denture base) have better fit and accuracy compared to the conventional RPDs?”

### 2.2. Search Strategy

In this systematic review, PubMed/MEDLINE, Cochrane collaboration, Science direct, and Scopus databases were searched for abstracts and full-text articles available online in July 2020 with no language restrictions. The included articles were searched on the basis of population or patients, including intervention, comparison, and outcome of study. The search was primarily focused on MeSH keywords: Denture, Partial, Removable, Dental Prosthesis, Denture Design, Denture framework, Removable partial denture framework, Removable partial denture designing, Printing, 3D and RPD, 3D printing and RPD, CAD/CAM and RPD, SLS and RPD printing, and SLM and RPD printing. The search was further expanded through scrutiny of references from included articles. Three investigators thoroughly obtained all relevant data and analyzed the results. Any disagreement between the investigators was resolved by consensus and discussion. All included articles assessed the accuracy of fit, retention, and stability of RPDs made with both conventional and digital techniques. After analyzing the data, the final full-text articles were selected, and any repeated studies were removed after reading the titles and abstracts.

### 2.3. Eligibility Criteria for Literature Search

Inclusion criteria are as follows: clinical trial studies involving comparison between accuracy and fit of RPD frameworks fabricated by conventional and digital techniques, published in English, with both in vitro and in vivo study designs; studies conducted with minimum of 04 dental casts were included; and studies with use of 3D dental cast scanners and 3D printing machines and milling techniques. Studies included analysis of RPD components in terms of fit and accuracy. Lastly, articles with both direct and indirect cast scanning methods were included.

The last search was performed in September 2020. In the 09 articles that were included, the casts or impressions were produced with intraoral scanners, CAD printing, and digital surveys. Fabrication of RPDs was performed using CAD/CAM software, SLS, SLM, SLA, indirect RP, and direct RP. The frameworks were fabricated by cobalt chromium alloys. Every included article was assessed, and the data were extracted based on the following parameters: authors name, study design, assessment methods, follow-up period, study groups, sample size, mean and STD, relevant features of study, and outcome, by three investigators (N.A, S.H and M.S.Z).

Exclusion criteria are as follows: studies that were literature reviews, clinical reports, surveys, or systematic reviews; studies including fixed prosthesis rather than removable partial dentures; studies not including comparisons between digital and conventional techniques; studies missing either one of the two techniques, i.e., conventional or digital; studies that did not focus on the fit of the denture; studies with incomplete information were excluded in this review.

### 2.4. Quality Assessment of Selected Studies

Furthermore, the quality assessment was assessed according to the parameters described in the Cochrane handbook for systematic reviews of interventions (v5.1.0) [11]. The same 3 review authors autonomously sort out the search to amplify the number of studies recovered. The reviewers surveyed every selected article for the predefined consideration criteria and directed impartial appraisals, and any ambiguity was settled by discussion and agreement or by consultation with a 4th reviewer (N.A). The Newcastle-Ottawa quality assessment scale (NOS) [12] was used for further analysis of the specific included articles.

## 3. Results

### 3.1. Results of Literature Search

**Figure 1:**
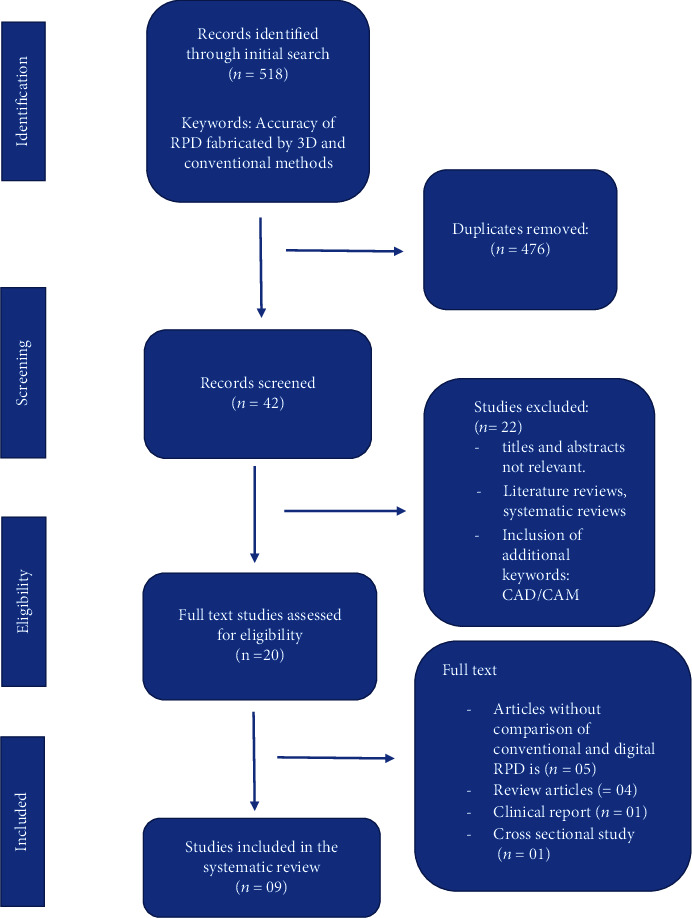
PRISMA flow diagram of systematic review.

The combined search identified 518 studies; out of which, 476 duplicate studies were removed, leaving 42 references for further evaluation. Irrelevant abstracts and titles were removed, and 20 full-text articles were assessed for eligibility. Nine studies [13–21] representing the relevant findings were included in the systematic review. The PRISMA flow diagram adopted in this systematic review is shown in Figure 1. Eleven full-text articles [13, 22–32] were excluded on the basis of lacking a comparability between digital and conventionally fabricated RPDs, review articles, cross-sectional study, and clinical reports (Table 1).

### 3.2. General Characteristics of Included Studies

All studies included were clinical trials, 04 in vivo trials [13, 14, 16, 19] and 5 in vitro trials [15, 17, 18, 20, 21]. One of the studies assessed the ease of cleaning, ability to speak, comfort, esthetics, stability, and ability to masticate [14]. Similarly, the retention of RPD framework constructed with traditional and digital workflow was compared in one study [16]. Seven studies evaluated the fit accuracy and analyzed the gap between denture framework and master cast, or tissue surfaces [13, 15, 17–21]. The number of study casts to analyze the fit and accuracy of RPD frameworks within the included studies ranged from 1 to 20. The follow-up period in the included studies varied from 1 to 6 months. Three of the studies contemplated the rest surfaces [15, 17, 18]. Cobalt chromium alloy was used in majority of studies to fabricate digital and conventional denture frameworks covered with acrylic resins. Polyetheretherketone (PEEK) was used in two articles to construct RPD frameworks. The general characteristic of included studies is summarized in Table 2.

### 3.3. Digital Method of Fabrication in Included Studies

Digital methods of fabrication of RPDs were used in these studies [13–21], each with a different type of CAD/CAM system. In one study [14], sintering laser technology was used to construct RPDs. SLM technique was applied in 5 studies [13, 15, 17, 19, 20] which proved to have a better fit accuracy than conventional lost wax technique; furthermore, for impression, making 1 study [19] partially used direct intraoral scan and CAD for fabricating RPDs, whereas indirect cast scanning with CAD was performed in all included research articles [13–21].

### 3.4. General Outcomes of Included Studies

The majority of the studies [14–20] concluded that RPDs constructed via digital workflow had a better fit, accuracy, and satisfaction of patients. Nevertheless, 2 studies [18, 20] found conventional RPD frameworks to be superior in terms of fit and accuracy in long-span RPDs (Table 3). The least clinical gap between RPD framework and tissue surface or cast was observed in CAD/CAM technique 41.677 ± 15.546 *μ*m. The fit discrepancy was 97.452 ± 32.575 *μ*m in RPDs constructed with RP (SLM and SLS) 3D printing. The RPD framework fabricated with conventional methods showed a higher mean framework fit discrepancy 114.063 ± 77.704 *μ*m. The fit and accuracy comparison of RPD frameworks fabricated with digital and conventional methods is presented in Table 4.

### 3.5. Results of Quality Assessment

Out of the 9 articles included, the randomization in subjects was performed in 4 articles [13, 14, 16, 19]. In 3 studies [13, 14, 19], blinding was carried out. One study [14] mentioned dropout rate of participants. The variables were measured several times in 7 of the included studies [13–19]. Sample size calculation was mentioned in 8 articles [13–19, 21]. Inclusion criteria were clearly mentioned in 4 studies [13, 14, 18, 20]. Furthermore, examiner reliability was tested in 5 of the studies [13, 15, 18–20]. The expected outcomes were prespecified in all included studies [13–21]. The results of quality assessment are stated in Table 5. Four studies [15, 16, 18, 20] fall in moderate bias category while 3 studies [13, 14, 19] had low risk of biasness, whereas 2 studies [17, 21] showed a high risk of biasness. In addition, “the quality assessment of selected studies on Newcastle-Ottawa quality assessment scale NOS [12] was ranging from 3 to 7 points.” Five studies [14, 16–19] were found to have a moderate risk of biasness. Four of the studies [13, 15, 20, 21] fall in high risk category. A mean score of 7.12 was achieved for the included studies (as mentioned in Table 6).

## 4. Discussion

In recent years, digital techniques such as CAD/CAM and RP systems have been used to fabricate RPDs. The digital technology offers many advantages, including precise planning of the denture frame components, reduced fabrication time, and improved functional and esthetic results, and improved quality of fit in RPD frameworks [26, 27]. Therefore, this systematic review was conducted to analyze and compare the fit accuracy of digital and conventionally fabricated frameworks/assemblies of RPDs. In this review, both in vitro and in vivo clinical trials were incorporated to get substantial evidence. The included studies used different types of digital techniques, materials, and assessment methods. In literature, one of the most reported manufacturing processes is CAD/CAM milling. However, RP is also gaining prominence recently specially with the introduction of newer techniques such as SLA, SLM, SLS, selective deposition modeling (SDM), fused deposition molding (FDM), and direct inkjet printing (DIP) [21]. Various studies showed promising results when the fit of RPDs fabricated with RP techniques were evaluated [14, 17, 20, 23]. However, in an in vitro study by Arnold et al. [17], RPDs fabricated with RP techniques showed distinct fitting irregularities, while RPDs fabricated with a milling technique showed significantly better framework fit as compared to the traditional ones. In majority of the studies, RPDs constructed via digital method had better accuracy of fit [13, 15, 19, 20] although no study discussed the long-term clinical performance. Furthermore, various methods have been reported in the literature to evaluate the fit and accuracy of RPD frameworks, including visual inspection, pressing test, color mapping, and indirect measurements of the gap filled with an impression material [9, 13, 22, 31].

Seven of the included studies [13, 15, 17–21] evaluated the fit accuracy of RPD frameworks. Soltanzadeh et al. [18] revealed that the conventionally fabricated RPD frameworks had a better fit accuracy compared to the 3D-printed frameworks; color mapping was carried out through comprehensive metrology software as an assessment tool. Similarly, Chen et al. [20] reported that conventional RPD frameworks showed better outcome in long span partially edentulous arches. However, a clinical gap of up to <0.2 mm can be achieved in short-span RPD frameworks fabricated with SLM methods. The remaining 5 studies revealed that the digitally fabricated RPD frameworks were more accurate than conventional ones. The studies used different assessment and fabrication methods.

In a study by Tregerman et al. [19], the framework was fabricated from a Co-Cr alloy by SLM. The evaluation consisted of scoring a survey with seven framework-related parameters and was completed by five clinicians, concluding that the sole digital method of fabrication was found to be the best [19]. Almufleh et al. [14] compared patient's satisfaction of RPDs fabricated by conventional and laser-sintering technology. More satisfaction was observed with the prosthesis fabricated with SLS technique. They reported that SLS-based RPD was more efficient, retentive, stable, and comfortable, and it improved their mastication and speech. This significant difference could be related to the enhanced mechanical properties of laser-sintered alloys, which are harder, denser, and proved a better microstructural organization with higher yield strength and ultimate tensile strength than cast cobalt chromium alloys, although the study had a small sample size and short follow-up that could limit the generalizability to long-term clinical performance.

In another clinical study by Maryod et al. [16], retention of digitally processed RPDs and conventional RPDs was evaluated. The results showed that the digitally processed RPDs were more retentive as it was associated with less human intervention. Mohamed et al. [23] carried out the clinical and cytological evaluation of RPDs fabricated by SLS additive prototyping technique and found a favorable oral environment along with an accurate fit and adaptation. Cytologically, at the microscopic level, no inflammatory cells were traced in the normally desquamated oral epithelial cells.

Although the result of the included studies was favorable for milling and 3D printing RPD frameworks, there were certain limitations to our study. For instance, most of the studies [13, 15, 17–20] did not contain a follow-up period. Except in a study by Almufleh et al. [14], a follow-up period was four weeks, and Waleed et al. evaluated the prosthesis after three months [16]. However, most of the studies were in vitro studies [15, 17–20], and the results were not correlated with in vivo investigations. Therefore, it was not clear that the digital RPDs fabricated in these studies are suitable for clinical application or not. Furthermore, in this review, three studies were lacking blindness [15–17]. Though one of the studies showed that the digitally fabricated RPDs had short term accuracy [14], similarly, Pooya et al. found that conventionally fabricated dentures had a better fit compared to digital RPDs [18]. To explore these shortcomings and improve the scientific evidence, we recommend further studies should be carried out with a larger sample size and more importantly with long-term follow-up periods and clinical correlation to predict the actual outcome and fit accuracy of conventional and digitally fabricated RPDs.

## 5. Conclusions

The results of this review described that the fit accuracy of removable partial denture frameworks fabricated by digital (CAD/CAM and RP) techniques is superior as compared to the frameworks fabricated by conventional techniques. According to the included studies in this review, the CAD/CAM and rapid prototyping RPD frameworks showed clinically acceptable gaps and fit accuracy in comparison to RPD constructed with conventional techniques. However, CAD/CAM and rapid prototyping techniques are increasing the scope of digital dentistry but are still under development. Further studies are required to assess their accuracy with clinical performance in removable prosthodontics.

## Figures and Tables

**Table 1:** Studies excluded from this review after full-text assessment and reason for exclusion.

Study	Reason of exclusion
Lee et al. [22]	No comparison with conventional dentures
Mohamed et al. [23]	No comparison with conventional dentures
Tang et al. [24]	No comparison with digital dentures
Jevremovic et al. [25]	Cross-sectional analytical studies
Pereira [26]	Systematic review
Biglin et al. [27]	Literature review
Abdulla et al. [28]	Literature review
Lin et al. [29]	Literature review
Harb et al. [30]	Clinical report
Negm et al. [31]	No comparison with conventional dentures
Tasaka et al. [32]	No comparison with conventional dentures

**Table 2:** General characteristics of selected studies included in the review.

Authors	Study design	RPD fabrication		Assessment method	Follow-up period	Outcome
		Study	Control			
Ye et al. [13]	In vivo, clinical trial	CAD and SLM techniques	LWT	Visual inspection and silicone fit checker	Nil	Clinically acceptable fit with CAD and SLM techniques
Almufleh et al. [14]	In vivo, cross over clinical trial	Period 1: casting and CAD/CAM laser sintered Period 2: CAD/CAM laser sintered	Period 1: laser sintered and casting Period 2: casting only	Nine-item based questionnaire	1, 2, and 4 weeks	Patient satisfaction was high with CAD/CAM sintering laser techniques
Bajunaid et al. [15]	In vitro, clinical trial	SLM technique	LWT	Silicone fit checker	Nil	The fit was accurate with SLM technique
Maryod et al. [16]	In vivo, crossover clinical trial	CAD/CAM	Conventional RPD	Digital force meter analysis	1 month and 3 months	CAD/CAM denture had higher retention
Arnold et al. [17]	In vitro, clinical trial	CAD/CAM and SLM	LWT	Light microscopy and stability check	Nil	Improved fit with CAD/CAM technique. SLM framework had discrepancies
Soltanzadeh et al. [18]	In vitro, clinical trial	CAD printing	LWT	Visual inspection	Nil	The conventional RPD framework was accurate
Tregerman et al. [19]	In vivo, clinical trial	Intra oral scanning SLM	Conventional impression, LWT, and casting	Yes/no rater scale by 02 general dentists and 03 prosthodontist	Nil	The complete digital method of RPD framework was superior
Chen et al. [20]	In vitro, clinical trial	SLM	LWT	Silicone fit checker	Nil	Fit and accuracy of SLM was accurate in small span denture base and retainer frameworks, while conventional RPD in large spans.
Honqqiang et al. [21]	In vitro, clinical trial	PEEK and CAM/CAM	LWT	Visual inspection, pressing test, and silicone fit checker	Nil	The fit of CAD/CAM, RPD was superior

RPD: removable partial denture; CAD/CAM: computer-aided designing and computer-aided manufacturing; 3D: three dimensional; SLM: selective laser melting; LWT: lost wax technique; PEEK: polyetheretherketone.

**Table 3:** Methodological analysis and main outcome of the selected studies.

Authors	Groups		Sample size		Mean and STD in *μ*m		Relevant features of study	Outcome
	Study	Control	Study	Control	Study	Control		
Ye et al. [13]	CAD and SLM	LWT	*N* = 15	*N* = 15	Zone C: 165 ± 112 Zone M: 180 ± 125 Zone R: 178 ± 123	Zone C: 108 ± 84 Zone M: 110 ± 93 Zone R: 104 ± 87	Compared the difference in gap thickness between different zones of (Co-Cr) frameworks	Clinically acceptable fit with CAD and SLM techniques
Almufleh et al. [14]	Casting, CAD/CAM, and laser sintered	Laser sintered and casting Casting only	*N* = 7 *N* = 5	*N* = 5 *N* = 4	63 ± 8	69.4 ± 14.9	The cobalt chromium alloy framework was compared in twelve participants. Dropout rate was 03 in this study	Patient satisfaction was high with CAD/CAM sintering laser techniques
Bajunaid et al. [15]	SLM technique	LWT	*N* = 15	*N* = 15	Short span 312.92 Long span 231.41	Short span 162.73 Long span 185.32	The measurements of the Co-Cr were determined at buccal, lingual, marginal, and central zones	The fit was accurate with SLM technique
Maryod et al. [16]	CAD/CAM	Conventional RPD	*N* = 20	*N* = 20	1. Retention: insertion visit 9.45 ± 1.76. 2. After 1 month 9.16 ± 1.71. 3. After 3 months 8.77 ± 1.61	1. Retention: insertion visit 19.02 ± 2.92 2. After 1 month 18.87 ± 2.92 3. After 3 months 15.99 ± 2.97	Digital force gauge was used for denture (Co-Cr) retention measurements up to 20 kg at different intervals	The retention (through retainer or clasp) of digitally fabricated dentures was higher than conventional dentures
Arnold et al. [17]	CAD/CAM and SLM	LWT	*N* = 12	*N* = 3	Indirect CAD/CAM horizontal arm 117 ± 34 Vertical arm 45 ± 21 Direct CAD/CAM horizontal arm 43 ± 23 Vertical arm 38 ± 21 Indirect RP horizontal arm 323 ± 118 Vertical arm 112 ± 60 Direct RP horizontal arm 365 ± 205 Vertical arm 363 ± 133	Mean LWT RPD horizontal clasp arm 133 ± 59 Vertical clasp arm 74 ± 25	Cobalt chromium frameworks were analyzed in laboratory with light microscope and stability testing	Improved fit with CAD/CAM technique. SLM framework had discrepancies
Soltanzadeh et al. [18]	CAD printing	LWT	*N* = 10	*N* = 10	Major connector: 0.19 ± 0.03 Rest 0.03 ± 0.03 Guiding plates 0.12 ± 0.03 Reciprocal plates 0.013 ± 0.04 Approaching arms 0.41 ± 0.06	Major connector 0.04 ± 0.08 Rest 0.02 ± 0.02 Guiding plates 0.03 ± 0.03 Reciprocal plates 0.0002 ± 0.05 Approaching arms 0.35 ± 0.2	Color mapping was used with metrology software. The gaps between denture framework and (trios 3) scanned models were analyzed at 8 points. The framework made of cobalt chromium alloy	The conventional RPD was accurate than 3D printed RPD.
Tregerman et al. [19]	Intra oral scanning SLM	Conventional impression, LWT and casting	*N* = 09	*N* = 09	The complete digital method was ratted better than analog and analog-digital methods (*p* < 0.001)	The complete digital method was ratted better than analog and analog-digital methods (*p* < 0.001)	The digital scan was carried out with TRIOS 3D shape. The quality and fit of Co-Cr frameworks were rated by clinicians	The digital RPD platform process was superior to the traditional and analog-digital approaches
Chen et al. [20]	SLM	LWT	*N* = 4	*N* = 4	Kennedy 1: 0.05 ± 0.0833. Kennedy 2: 0.1 ± 0.0641. Kennedy 3: −0.0033 ± 0.0873 Kennedy 4: −0.1467 ± 0.035	Kennedy 1: −0.0133 ± 0.0665. Kennedy 2: −0.05 ± 0.0641. Kennedy 3: 0.12 ± 0.0234 Kennedy 4: −0.0567 ± 0.0577	Cobalt chromium frameworks on 4 resin-based Kennedy's arches were constructed	Regardless of the production method, a good fit was achieved between the frameworks, “connector, clasp” and the corresponding resin models
Honqqiang et al. [21]	PEEK and CAM/CAM	LWT	*N* = 15	*N* = 15	42.8 ± 29.4	130.9 ± 50.5	One-piece PEEK RPDs were formed by the CAD/CAM and LWT	The fit of CAD/CAM and RPD was superior

CLW: conventional lost wax technique; RPD: removable partial denture; CAD/CAM: computer-aided designing and computer-aided manufacturing; 3D: three dimensional; SLM: selective laser melting; LWT: lost wax technique; PEEK: polyetheretherketone; STD: standard deviation.

**Table 4:** Comparison of fit and accuracy of RPD frameworks constructed with conventional and digital techniques.

Digital framework	Material	Fit accuracy (*μ*m)	
		Mean	SD
CAD/CAM	Co-Cr alloy and PEEK	41.677	15.546
SLM/SLS	Co-Cr alloy	97.452	32.575
Conventional framework			
Lost wax technique	Co-Cr alloy	114.063	77.704

Co-Cr: cobalt chromium; PEEK: polyetheretherketone; CAD/CAM: computer-aided designing and computer-aided manufacturing; SLM: selective laser melting; SD: standard deviation; SLS: selective laser sintering; *μ*m: micrometer; RPD: removable partial denture.

**Table 5:** Methodological quality assessment results of the selected studies according to the standards described in the Cochrane handbook for systematic reviews of interventions (v5.1.0).

Study	Patient chosen randomly	Blinding		Withdrawal/dropout mentioned	Variables measured many times	Sample size	Inclusion/exclusion criteria clear	Examiner reliability tested	Clearly report all expected outcomes prespecified	Quality of study/bias risk
		Participants	Assessor							
Ye et al. [13]	Yes	Unclear	Yes	No	Yes	Yes	Yes	Yes	Yes	Low
Almufleh et al. [14]	Yes	Yes	Yes	Yes	No	Yes	Yes	No	Yes	Low
Bajunaid et al. [15]	Unclear	No	No	No	No	Yes	No	Yes	Yes	Moderate
Maryod et al. [16]	Yes	No	No	No	No	Yes	No	No	Yes	Moderate
Arnold et al. [17]	Unclear	No	No	No	No	Yes	No	No	Yes	High
Soltanzadeh et al. [18]	Unclear	No	No	No	Yes	Yes	Yes	Yes	Yes	Moderate
Tregerman et al. [19]	Yes	Unclear	Yes	No	Yes	Yes	No	Yes	Yes	Low
Chen et al. [20]	Unclear	No	No	No	Yes	No	Yes	Yes	Yes	Moderate
Honqqiang et al. [21]	Unclear	No	No	No	No	Yes	No	No	Yes	High

^∗^A study was graded to have a low risk of bias if it yielded 6 or more “yes” answers to the 9 questions, moderate risk if it yielded 3 to 5 “yes” answers, and high risk if it yielded 2 “yes” answers or less.

**Table 6:** Newcastle-Ottawa quality assessment of selected studies.

Author ID year	Selection	Comparability	Exposure	Total
Ye et al. [13]	^∗∗^	^∗^	^∗∗^	5
Almufleh et al. [14]	^∗∗^	^∗^	^∗∗∗∗^	7
Bajunaid et al. [15]	^∗∗^	^∗^	^∗∗^	5
Maryod et al. [16]	^∗∗∗^	^∗^	^∗∗^	6
Arnold et al. [17]	^∗∗^	^∗^	^∗∗∗^	6
Soltanzadeh et al. [18]	^∗∗∗^	^∗^	^∗∗^	6
Tregerman et al. [19]	^∗∗^	^∗^	^∗∗∗^	6
Chen et al. [20]	^∗∗^	^∗^	^∗∗^	5
Honqqiang et al. [21]	^∗^	^∗^	^∗^	3

^∗^A study can be awarded a maximum of 1 star for each numbered item within the selection and exposure categories. A maximum of 2 stars can be given for comparability. Each study can be awarded a total of 9 stars. A study was rated to have a low risk of biasness if it received the maximum allowed number of 9 “stars” while moderate risk if it received 8, 7, or 6 “stars” and high risk if it received 5 “stars” or less.

## Data Availability

The raw data used to support the findings of this study are included within the article.
